# Uptake of minimum acceptable diet among children aged 6–23 months in orthodox religion followers during fasting season in rural area, DEMBECHA, north West Ethiopia

**DOI:** 10.1186/s40795-019-0274-y

**Published:** 2019-02-27

**Authors:** Efram Mulat, Girma Alem, Wubetu Woyraw, Habtamu Temesgen

**Affiliations:** 1 Dembecha Health Center, WEST Gojam Zone Health Department, Dembecha, Ethiopia; 2 Department of Nurse, College of Health Sciences, Debre Markos University, P.O. Box 269, Debre Markos, Ethiopia; 3 Lecturer of Nutrition, Department of Human Nutrition and Food Sciences, College of Health Science, Debre Markos University, P.O. Box 269 Debre Markos, Ethiopia

**Keywords:** Minimum acceptable diet, Fasting, Orthodox, Dietary diversity, Meal frequency

## Abstract

**Background:**

Under-nutrition is the cause for poor physical and mental development and has more burden among infants and young children aged between 6 and 23 months. Cultural practices like not providing animal source foods for infants and young child aged between 6 and 23 months were barrier for practicing proper children feeding. The aim of this study was to assess minimum acceptable diet and associated factors among children aged between 6 and 23 months in Orthodox religion during fasting season in rural area, Dembecha, Ethiopia.

**Methods:**

A community-based cross-sectional study was conducted to assess Minimum Acceptable diet.

Random sampling technique was applied to select 506 study participants. Interview was used to collect data on Practice of minimum acceptable diet, minimum dietary diversity, minimum meal frequency and related factors among children aged between 6 and 23 months from mothers / caregivers.

**Result:**

About 8.6% of infants and young children aged between 6 and 23 months received minimum acceptable diet. Education status of mother(AOR = 0.22,95%CI:0.1, 0.48), involvement of mother in decision making (AOR = 0.22,95%CI:0.10,0.48), birth order of index children (AOR = 0.36,95%CI:0.14,0. 94), knowledge on feeding frequency (AOR = 0.3,95% CI:0.16,0.58), and institutional delivery (AOR = 5.13, 95%CI: 1.26, 20.80) were significantly associated with minimum acceptable diet.

**Conclusion:**

Minimum acceptable diet practice was low. Educational status of mother, involvement of mother in decision making, knowledge on feeding frequency and institutional delivery were significantly associated with minimum acceptable diet. This indicates that nutrition education and counseling related to infant and young child feeding practice is not addressed for all mothers. Strengthening mothers’ education on acceptable child feed practice, and working with religion leaders to increase knowledge of mothers on child feed practice are recommended.

**Electronic supplementary material:**

The online version of this article (10.1186/s40795-019-0274-y) contains supplementary material, which is available to authorized users.

## Background

Inappropriate feeding practice of Infants and young child leads to malnutrition, this exposes the children to under nutrition, increasing morbidity and mortality, and chronic stunting that will be continuing to next generations [1–3]. By improving the quality and frequency of complementary feeding practice it is possible to improve health, reducing morbidity and mortality of young children [4]. Nearly one third of children deaths could be prevented by appropriate complementary feeding practices [5, 6]. Early initiation of breastfeeding, exclusive breastfeeding, implementing complementary feeding, consumption of diversified diet, adequate meal frequency, and consumption of iron fortified foods are core indicators for monitoring feeding practices of infants and children. Minimum Acceptable diet defined by WHO as is the proportion of children 6–23 months of age who receive a minimum acceptable diet (both minimum dietary diversity and the minimum meal frequency) during the previous 24 h [6, 7].

In developing countries including Ethiopia, feeding infants and children with diversified diet is practiced inappropriately. In Africa less than one-third and one-half of children aged between 6 and 23 months met the minimum criteria for dietary diversity and meal frequency, respectively. That is why the prevalence of stunting in African countries increases threefold during the first 2 years of life [8].

Burden of stunting is high in Amhara region and children in rural are more vulnerable for stunting than urban children [9, 10]. Minimum dietary diversity practice is low in Amhara region than national i.e., 2% of children in Amhara region got minimum dietary diversity. Children of Orthodox mother were 40% less likely to receive minimum dietary diversity than non-Orthodox children [11]. In our country, especially in rural area, there is poor practice in children feeding [9, 12].

Around 50- 7 % of death in under-five children in Ethiopia is accounted by inappropriate complementary feeding practices [4]. Malnutrition below 2 years of age leads children to become vulnerable to growth retardation, delayed mental development, micronutrient deficiencies, and common childhood illnesses and death. [13]. Culture has effect on practice of dietary diversity, meal frequency and minimum acceptable diet.

According to Ethiopian demographic health survey data analysis, consumption of animal source by orthodox religion followers children were less than other religion follower families [11].

Both minimum dietary diversity and meal frequency practices were affected by age of a child, birth order of index child, religion, and media exposure of mother. Minimum dietary diversity separately affected by educational level of a mother, residence and home gardening activity while mother’s involvement in household decision making and postnatal visit have significant association with minimum meal frequency [11, 12, 14–20].

Minimum acceptable diet indicator is used for assessing infant and young child feeding practices and it is a composite indicator comprises minimum dietary diversity and minimum meal frequency indicators and to measure both quality and quantity of nutrients [5, 7]. A cross- sectional study conducted in primary health care facility in 2015 to assess magnitude of minimum acceptable diet in Indonesia found that 66.6% of infants and young children aged 6–23 months consume minimum diversified diet [21]. A community- based cross-sectional study conducted in 2016 among rural resident in Nigerian Infant and Young Children (IYC) showed that 31.5% infants consumed the minimum dietary diversity. [22].

Another community-based cross-sectional study conducted in southern part of Ethiopia (Arsi Zone) in 2015 showed that only18.8% of children aged between 6 and 23 months consumed four or more food groups to meet the minimum dietary diversity criteria [23]. A health institution based cross sectional-study conducted in Addis Ababa in 2016 showed that 59.9% of the children aged 6–23 months meet the minimum requirement of diversified diet [15].

A cross-sectional study done to assess dietary diversity and meal frequency feeding practice in wailayita sodo town in 2015 showed that only 27.3% of infants and young children consumed diversified diet [24]. Studies conducted in different parts of Amhara region, North West Ethiopia, showed that low practice of feeding children with diversified diet [12, 25]. Study conducted in Northwest Ethiopia in 2016 to assess weaning practice and associated factors in Feres Bete town indicate that about 43.9% of infants and young children met minimum diversified diet [26].

Community- based cross -sectional study conducted in Northwest Ethiopia in 2015 to assess dietary diversity, meal frequency and associated factors among IYC showed that around 50.4% of children received minimum meal frequency [12]. The same study conducted in wolaita Sodo town in 2015 showed that 68.9% of IYC met the minimum meal frequency effect [24].

A healthy care facility based cross-sectional study conducted in Indonesia in 2015 to determine minimum acceptable diet showed that only 47.7% of children met the minimum acceptable diet. [21]. A result from analysis of 10 Sub Saharan countries’ demographic and health surveys between 2010 and 2013 revealed that 18% of children aged 6–23 months met minimum acceptable diet. The surveys’ analysis also suggested that women’s empowerment enhance practice of infant and young children MAD. Greater overall empowerment of women was consistently and positively associated with multiple IYCF practices in Mali, Rwanda and Sierra Leone, but negative relationships were found in Benin and Niger. Null or mixed results were observed in the remaining countries [27].

Nutrition education has a great role to improve food security and this enhance to improve nutritional status of children using diversified diet feeding for children [16, 28]. Mother education is one determinant factor affecting practice of feeding diversified food. Children born from mothers who were well educated and had a secondary level education or higher education had feeding with diversified foods [12, 15]. Media exposure and income level have positive association with dietary diversity practice. This showed that media exposure has increased the chance infant and young child to feed with diversified food [12]. Children in houses of having high level of income had consumed highly diversified diet than those living in a house of low income level [15, 25].

Involvement of mothers in household decision found to be another determinant factor affecting practice of minimum meal frequency [12, 18]. As a result children aged 6–23 months will not consume recommended minimum acceptable diet in fasting season. As indicted above variety of studies was conducted and come up with different results of minimum acceptable diet. But none of them considers fasting season in orthodox religion followers.

Therefore this study is needed to assess practice of minimum acceptable diet and to identify associated factors. Although the study area is the area where variety of foods is available, there is no study conducted in Dembecha district on practice of minimum acceptable diet.

## Methods

### Study design and setting

A community-based cross-sectional study was conducted to assess minimum acceptable diet and factors affecting minimum acceptable diet practice among infants and young children aged 6–23 months during fasting season in rural areas of Dembecha district, 2018. Dembecha district is located at 349 km North West from Addis Ababa, and 203 km from Bahir Dar, capital city of Amhara region. Greater than 95% of the population is orthodox religion followers and the rest are other religion followers. The total numbers of children under 5 years is 21, 571 (Dembecha district health office, annual report, 2018). The study was conducted from February 25 to March 27, 2018.

### Participants

Infants and young children aged 6–23 months with mothers / caregivers who were orthodox religion followers and resident of Dembecha district for the past 6 months and available during data collection period were included. Participants were selected using random sampling technique. Among the total kebeles seven kebeles were selected randomly and the total sample size was allocated proportionally. Finally study participants were selected systematically from health post registers. Infants and young children aged 6–23 months with acute illness and other conditions that disturb appetite during data collection period and infants and young children with mothers/caregivers who were seriously ill and cannot give response did not participated in the study. The sample size was calculated using single population proportion considering the proportion of minimum acceptable diet as 12.3, 95% CI, 3% of margin of error, and 10% non-response rate. The proportion is taken from study conducted to assess complementary feeding practice in 2015 in Arsi district, southern Ethiopia [23].

The single proportion formula is stated as: $\mathrm{n}=\frac{{\left(Z\frac{\alpha }{2}\right)}^2\mathrm{p}\left(1-p\right)}{(d)^2}$

$$ \mathrm{n}1=\frac{(1.96)^{2\kern1.25em }0.123\;\left(1-0.123\right)}{(0.03)^2}=460 $$

$$ \mathrm{N}1=\frac{460+460\times 10}{100}=506 $$

The final sample size was 506.

Where

- P = proportion of minimum acceptable diet
- Za/2 = critical value at confidence interval
- d = margin of error

**Fig. 1:**
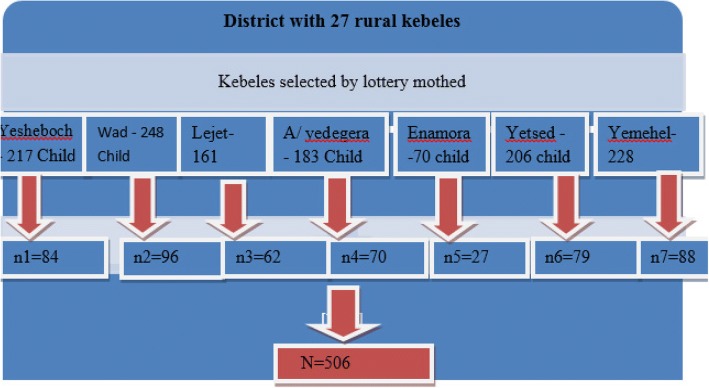
Schematic presentation of sampling procedure from each selected kebels, Dembecha, North -West Ethiopia, 2018

Random sampling technique was used to select children aged between 6 and 23 months. Infants and young children aged between 6 and 23 months with their mothers were registered in health post. The registration was used as sampling frame (Fig. 1).

## Study variable

### Independent variables

The independent variables for this study were maternal/caregiver’s demographic and socio-economic characteristics(ethnicity, income, education, age, occupation, marital status), child age, child sex, birth order, maternal knowledge on minimum acceptable diet practice, training, education given on IYCF, media exposure,(antenatal care, postnatal care, Growth monitoring and promotion service, vaccination service.

Satisfactory exposure to media: Women aged 15–49 years at least once a week who listen to radio, or watch television.

Educated: those parents of infants and young children aged between 6 and 23 months who can read and write.

None educated: parents of infants and young children aged between 6 and 23 months who cannot read and write (Additional file 1).

### Dependent variable

#### Minimum acceptable diet was the dependent variable of this study

Minimum dietary diversity: Proportion of children aged 6–23 months who received four or more food groups from the seven food groups during the previous 24 h.

Minimum meal frequency: Proportion of breastfed and non-breastfed children aged 6–23 months who received solid, semisolid, or soft foods (but also including milk for non-breastfed children). The Minimum frequency will be defined as: at least twice for breastfed infants 6–8 months, at least three times for breastfed children 9–23 months, and at least four times of foods and 2 feeding of milk products for non-breastfed children 6–23 months during the previous 24 h.

Minimum Acceptable diet: Proportion of children 6–23 months of age who receive a minimum acceptable diet (both minimum dietary diversity and the minimum meal frequency) during the previous 24 h.

*Milk feeding frequency for non*-*breasted child* is proportion of non-breastfed children 6–23 months of age who receive at least two times of milk feeding (Additional file 1).

#### Data collection procedures

Primary data on Practice of minimum acceptable diet, minimum dietary diversity, minimum meal frequency and related factors were collected from mothers or caregivers who had child aged 6–23 months and follower of orthodox religion followers. The data collection tool regarding the various dietary diversity and meal frequency was adopted from WHO IYCF indicator assessment tool with some modification to fit with the context. Household conditions, water source, electricity, livestock, agricultural products, farm land, income were collected as household wealth assets collected by using WFP tools of wealth index. Socio-demographic and other part of data collection tools were adopted from EDHS (2016) and prepared by investigators. Seven health extension workers and two public health officers were participated in data collection. Data were collected using interviewer-administered questionnaire.

### Data analysis and quality management

To assure high quality of the data, Training was given for data collectors and supervisors for **1** day. The questionnaire was tested on 5% of mothers who were not included in the study before the main data collection procedure began to enable the analysis to be conducted in a right way. The collected data was coded, entered and cleaned using Epi-data version 3.1 and was exported to SPSS version 20 for analysis. Frequencies and cross tabulation were calculated to describe the study population in relation to dependent variable. Logistic regression analysis was conducted to show the relationship between dependent and independent variables. Variables with *p*-value less than 0.25 in binary logistic regression were interred to multivariable logistic regression to identify independent effect of independent variables on dependent variables. Principal component analysis was conducted for categorizing household wealth status into three categories; poor, middle and rich.

## Result

### Socio-demographic characteristics of study population

A total of 502 infants and young children aged between 6 and 23 months with mother were enrolled in the study that makes a response rate of 99.2%. Among children of mother/caregivers, 259(51.6%) children were males and 171(34.1%) were categorized in the age between 18 and 23 months. The mean age of children was 14.5 ± 5.28 (SD) months. Of the mother/caregivers of children, 271(54%) had no education. Among study participants, 487(97%) mothers were married. About 325(64.7%) fathers of the children were educated. About 475(94.6%) of mothers were farmers (Table 1).

**Table 1:** Parental level socio demographic characteristic of infants and young children aged 6–23 months, Dembecha, North West Ethiopia, 2018

Characteristics	Frequency(n)	Percentage (%)
Mother age(years)		
15–24	91	18.1
25–34	295	58.8
35–49	116	23.1
Mother marital status		
Currently married	487	97.0
Separated	10	2.0
Divorced	5	1.0
Ethnicity		
Amhara	502	100.0
Mother educational status		
Not educated	271	54.0
Educated	231	46.0
Father educational status		
Non educated	177	35.3
Educated	325	64.7
Mother occupation		
Farmer	475	94.6
Housewife	16	3.2
Merchant	6	1.2
Daily worker	5	1.0
Father occupation		
Farmer	474	94.4
Employed	12	2.4
Daily worker	12	2.4
Merchant	4	0.8
Number of Under five children		
One	362	72.1
Two	136	27.1
Three	3	0.8
Family size the house		
Two-four	151	30.1
Five-six	228	45.4
Seven- ten	123	24.5
Age of children		
6–11(months)	169	33.7
12–17(months) 18–23 months	162 171	32.3 34.1
Sex of children		
Male	259	51.6
Female	243	48.4
Birth order of index child		
First	87	17.3
Second to third	146	29.1
Fourth to fifth	197	39.2
Six and above	72	14.3
Decision making		
Mothers not involved	270	53.8
Mothers involved	232	46.2
Wealth status		
Poor	150	29.9
Middle	186	37.1
Rich	166	33.1

### Child and mother health care utilization related characteristics

During pregnancy, 292(58.2%) of mother received focused ante-natal care service four and above times. Greater than two-third, 407(81.1%) children were delivered in health facility. Greater than half, 317(63.1%) of mothers did not received any post natal care. Nearly two-third, 356(70.9%) of children did not received growth monitoring and promotion service monthly. About 75 % of children were fully vaccinated. From all children, 497(99.0%) of children were breastfed (Table 2).

**Table 2:** Child and mother health care related characteristics of children aged between 6 and 23 months, Dembecha, North-West Ethiopia, 2018

Characteristics	Frequency(n)	Percentage (%)
Antenatal service		
Fourth and above times	292	58.2
One to three times	199	39.62
No follow up	11	2.2
Place of delivery		
Health facility	407	81.1
Home	95	18.9
Post natal care		
No follow up	317	63.1
Within three to six days	37	7.4
Growth monitoring and promotion service		
No	356	70.9
Yes	146	29.1
Children vaccination status		
Finished	381	75.9
Started	121	24.1
Current breast feeding status		
Breast feeding	497	99.0
Non breast feeding	5	1.0
Time of starting complementary food		
After six months	226	45.0
At six months	214	42.6
Before six months	62	12.4

### Education, knowledge on IYCF and fasting related characteristics

Greater than two-third, 449(89.4%) of mothers/caregivers of children had got education on dietary diversity and meal frequency. From mothers of children, 488(97.2%) mothers had knowledge on feeding children with diversified diet, and 75(14.9%) of mothers know frequency of feeding based on child age. Greater than half, 265(52.8%) of mothers were trained on food preparation demonstration with diversified diets. Greater than 334(66.5%) of children were living in houses where family member fed at any time of fasting season. Less than one third, 81(16.1%) of children were living with family member who fed animal source foods. About 69(13.7%) of mothers had satisfactory media exposure (Table 3).

**Table 3:** Education, media exposure, and fasting related characteristics of houses of children aged between 6 and 23 months, Dembecha, North West Ethiopia, 2018

Characteristics	Frequency(n)	Percentage (%)
Read newspapers and magazine		
No	486	96.8
One and above times a week	16	3.2
Education on dietary diversity and meal frequency		
Yes	449	89.4
No	53	10.6
Food cooking demonstration training		
Yes	265	52.8
No	237	47.2
Media exposure		
Unsatisfactory	433	86.3
Satisfactory	69	13.7
Knowledge on child feeding		
On dietary diversity	488	97.2
On feeding frequency	75	14.9
Reason for not feed animal source food		
Contaminate feeding utensils	143	28.4
Not necessary for children	48	9.5
Due to fasting	29	5.7
Family member feeding practice in fasting		
Not consume animal source foods	421	83.9
Consume at any time in fasting	334	66.5
Not consume at any time in fasting	168	33.5
Consume animal source foods	81	16.9

### Practice of minimum acceptable diet

From all children, 487(97.0%) of children received cereals and tubers. About 63 % of children received minimum meal frequency and 49(9.8%) children fulfill minimum acceptable diet. About 9.8% of children received minimum dietary diversity (Table 4).

**Table 4:** Practice of minimum acceptable diet, minimum dietary diversity and minimum meal frequency among children aged between 6 and 23 months, Dembecha, North - West Ethiopia, 2018

Food groups	Frequency(n)	Percentage (%)
Cereals & roots	487	97.0
Legumes & nuts	409	81.5
Other fruits and vegetables	257	51.2
Vitamin A rich fruits and vegetables	51	10.2
Eggs	45	9.0
Milk & its products	27	5.4
Flesh food	1	0.2
Minimum dietary diversity		
Non adequate	453	90.2
Adequate	49	9.8
Minimum meal frequency		
Adequate	319	63.5
Non adequate	182	36.3
Minimum acceptable diet		
Non adequate	459	91.4
Adequate	43	8.6

### Factors influencing minimum acceptable diet practices

Education status of mother(AOR = 0.22,95%CI:0.1, 0.48), involvement of mother in decision making (AOR = 0.22,95%CI:0.10,0.48),birth order of index children (AOR = 0.36,95%CI:0.14,0. 94), knowledge on feeding frequency (AOR = 0.3,95% CI:0.16,0.58), and institutional delivery (AOR = 5.13, 95%CI: 1.26, 20.80) were significantly associated with minimum acceptable diet (Table 5).

**Table 5:** A bivariate and multivariate logistic regression output showing factors associated with minimum acceptable diet practice among children aged between 6 and 23 months, Dembecha, North West Ethiopia, 2018

Characteristics	Minimum acceptable diet				
	Inadequate N (%)	Adequate N(%)	COR (95%)	AOR (95%)	*P*-Value
Mother education					
Educated	207 (89.6)	24 (10.4)	1	1	1
Non educated	252 (93.0)	19 (7.0)	0.65 (0.35,1.22)	0.44 (0.23, 0.86)	**0.01** ^*^
Father education					
Educated	293 (90.2)	32 (9.8)	1	1	1
Not educated	166 (93.8)	11 (6.2)	0.60 (0.29, 1.23)	0.7 (0.32, 1.57	0.4
Decision making in house					
Mothers involved	198 (85.3)	34 (14.7)	1	1	1
Mothers not involved	261 (96.7)	9 (3.3)	0.2 (0.09,0.43)	0.22 (0.1, 0.48)	0.0*
Birth order of index child					
First Fifth and above	130 (86.7)	20 (13.3)	1	1	1
Second to fourth	248 (93.6)	17 (6.4)	0.45 (0.23, 0.88)	0.28((0.14,0.54)	0.00*
First	81 (931)	6 (6.9)	0.48 (0.18, 1.25)	0.36 (0.14,0.94)	0.036*
Place of delivery					
Home	94 (98.9)	1 (1.1)	1	1	**1**
Health facility	365 (89.7)	42 (10.3)	10.82 (1.47, 79.61)	5.13 (1.26, 20.8	0.02*
Knowledge on MF					
Yes	62 (82.7	13 (17.3)	1	1	**1**
No	397 (93.0)	30 (7.0)	0.36 (0.17, 0.72)	0.3 (0.16, 0.58)	0.00*
Education given on MAD					
Yes	407 (90.6)	42 (9.4)	1	1	1
No	52 (98.1)	1 (1.9)	0.18 (0.03,1.38)	0.59 (0.07, 5.12)	0.63
Food preparation demonstration training					
Yes	232 (87.5)	33 (12.5)	1	1	1
No	227 (95.8)	10 (4.2)	0.31 (0.15,0.64)	0.48((0.22, 1.05)	0.06
Meeting of women health development army					
No	428 (92.0)	37 (8.0)	1	1	1
Yes	31 (83.8	6 (16.2)	2.24 (0.88, 5.71)	0.69 (0.21, 2.27)	0.54
Media exposure					
Satisfactory	67 (97.1)	2 (2.9)	1	1	1
Not satisfactory	392 (90.5)	41 (9.5)	3.5 (0.83 (14.83)	1.23 (0.52, 2.87)	**0.65**
Family member who ate at any time in fasting season					
Yes	299 (89.5)	35 (10.5)	1	1	1
No	160 (95.0)	8 (5.0)	0.43 (0.19, 0.94)	0.78 (0.32, 1.9)	0.59
Family member who consumed animal source foods					
No	404 (96.0)	17((4.0)	1	1	1
Yes	55 (67.9)	26 (32.1)	11.23 (5.73,22.0)	5.64 (2.8, 11.37)	0.00*
Believe on not feed child animal source foods					
Not believe	246 (87.2)	36 (12.8)	1	1	1
Believe	213 (96.8)	7 (3.2)	0.23 (0.098, 0.52)	0.53 (0.21,1.32)	0.17

Notice: * significant associated, *COR* Crude odds ratio, *AOR* Adjusted odds ratio. PV: *p*-value

## Discussion

. The study revealed that about 8.6% of children received minimum acceptable diet. This result is lower than findings of studies conducted in southern and northern part of Ethiopia which was 12.3 and 12.0%, respectively [23, 29, 30]. But the above mentioned studies determined minimum acceptable diet practice when they studied minimum dietary diversity and minimum meal frequency practiced without consideration of fasting. The difference might be due to high non educated mothers were included in this study but higher number of participants of the above three studies were educated. Difference in data collection period and the above studies include different religion followers who were not in fasting. Finding of this study is compatible with results i.e.,7.3 and 7% obtained from studies conducted in Nigeria and Ethiopian Demographic and health survey respectively [9, 22].

There is no study conducted to show the association between practice of minimum acceptable diet and associated factors in Ethiopia even if the practice was determined when minimum dietary diversity and minimum meal frequency were assessed. Therefore it is not possible to compare factors associated with minimum acceptable diet practice in this study with other study findings. Mother educational status was significantly associated with minimum acceptable diet. According to this study children born from mother who had education were 56% more likely to receive minimum acceptable diet when compared with children born from non-educated mother(AOR = 0.44,95%CI:0.23, 0.86). This showed that education enables mothers/caregivers to know benefits of practice of dietary diversity and feeding frequency and minimum acceptable diet practice in infants and young children aged between 6 and 23 months was adequate.

This study found that birth order of the index child is one of the predictors of practice of minimum acceptable diet. Children born in first order (AOR = 0.36, 95%CI: 0.16, 0.58), and second to fourth order (AOR = 0.28, 95%CI: 0.14, 0.54) were 64 and 72% less likely to receive minimum acceptable diet when compared with children born in above fourth order respectively. As parity of mother’s increases, there is high exposure to practice and information on diversified diet preparation and feeding frequency that enables children to meet minimum acceptable diet. Another important factor significantly associated with minimum acceptable diet during fasting season was mothers’ participation in household decision making. Children born from mothers involved in household decision making were 78% more likely to receive minimum acceptable diet when compared with children of mothers who were not involved in decision making(AOR = 0.22. 95%CI: 0.1,0.48). involvements of mothers in decision making in the household enables mothers to access money for purchasing foods which increases availability of some foods like egg and fruits and vegetables in the household. Then children would meet minimum acceptable diet. Delivery in health facility found to be significant associated with practice of minimum acceptable diet. Children born in health facility were 5 times more likely to receive minimum acceptable diet than who were born at home (AOR = 5.13, 95%CI: 1.26, 20.8). Mother counseling on child feeding after delivery in health facility increases knowledge of mother on dietary diversity and feeding frequency.

Other factor associated with minimum acceptable diet feeding practice was previous knowledge on feeding frequency based on age. Children whose mothers or caregiver’s did not have knowledge on feeding frequency (AOR = 0.3, 95%CI: 0.16, 0.58) were 70% less likely to receive minimum acceptable diet when compared to children of mothers who had knowledge of feeding frequency. This indicate that knowing feeding frequency based on child age leads the mother/ care givers to give diet timely throughout all seasons.

Presence of family member who fed animal source foods found to be significantly associated with practice of minimum acceptable diet. Children lived with family member who consumed animal source foods were 5 times more likely received minimum acceptable diet than those children who were living in houses with no family member who could consume animal source foods(AOR = 5.64, 95% CI: 2.8, 11.37). This showed that animal source foods might be prepared and its availability would be increased if consumer of this food group was present in the house. This would enable the child to consume animal source foods and this increases the number of food groups the child could fed.

Only 9.8% of children received minimum dietary diversity which is lower than results of similar study conducted in North West Ethiopia [31]. The difference might be due to participants’ residential difference. Studies conducted in southern part of Ethiopia that is 23.3% of children received foods from four and above food groups [14]. The difference might be due to high purchasing capacity and greater than 60% of mothers of the children were protestant religion followers who are not participated in fasting season. Due to this reason the practice of dietary diversity is high in the above study. Other study conducted in North West Ethiopia showed that 12.6% of children received minimum dietary diversity feeding which is greater than the result of this study [12]. The difference is due to educational status difference. About 58% of mothers had education in the above study, but 46% of mothers included in this study were educated. The result of this study showed that the practice of dietary diversity is higher than result of study conducted in other area of North West Ethiopia which was 8.5% [25]. The difference might be due to recent expansion of nutritional programs working in child nutrition that enables improvement in dietary diversity in rural area. In this study 63.5% of children fulfill the WHO minimum meal frequency criteria in 24 h. The result is lower than results of two studies conducted in southern part of Ethiopia with results of 67.3 and 68.9% respectively [23, 24]. The difference might be due to difference in religion status of mothers of children. But the result is higher than result of studies conducted in Ethiopia with result of 50.5%, and in North West Ethiopia with a result of 50.4% [12, 29]. The difference in results might be due to presence of low attention for child feeding in previous year.

## Conclusion

Minimum acceptable die practice in infants and young children aged between 6 and 23 months is low. Educational status of mother, involvement of mother in decision making, First birth order of index child, second to fourth birth order of index child, knowledge on feeding frequency were factors negatively associated with minimum acceptable diet. Delivery in health facility, and presence of family member who fed animal source foods during fasting were positively associated with minimum acceptable diet. Strengthening mothers’ education on acceptable child feed practice and working with religion leaders to increase knowledge of mother on child feed practice during fasting season. Conduct study with strong study design in wide area to determine minimum acceptable diet and factor associated with minimum acceptable diet practice.

## Additional file

Additional file 1: Questionnaire prepared for assessment of minimum acceptable diet among Infants and young children aged between 6-23 months in Fasting season, Dembecha, North -West Ethiopia, 2018 (DOCX 70 kb)

## Abbreviations

ANC: Antenatal care
DHS: Demographic and Health Survey
EDHS: Ethiopian Demographic and Health Survey
HSDP: Health Sector Development Plan
IYC: Infant and young child
IYCF: Infant and young child feeding
IYCMAD: Infant and young child minimum acceptable diet
IYCMDD: Infant and young child minimum dietary diversity
MAD: minimum acceptable die
MDD: minimum dietary diversity
MDG: millennium Development goal
MMF: minimum meal frequency
NGO: Non-Governmental Organization
PNC: post natal care
WFP: World Food Program
WHO: World Health Organization
