# Supplementary material for: Uptake of minimum acceptable diet among children aged 6–23 months in orthodox religion followers during fasting season in rural area, DEMBECHA, north West Ethiopia
Source: BMC Nutr. 2019 Feb 27;5:18. doi: 10.1186/s40795-019-0274-y (PMC7050747; doi:10.1186/s40795-019-0274-y)
Supplement: Supplementary file 1 — Questionnaire prepared for assessment of minimum acceptable diet among Infants and young children aged between 6-23 months in Fasting season, Dembecha, North -West Ethiopia, 2018 (DOCX 70 kb) [file 40795_2019_274_MOESM1_ESM.docx]

DEBRE MARKOSUNIVERSITY, COLLEGE OF HEALTH SCIENCE

Questionnaire

Questionnaire prepared for assessment of minimum acceptable diet among Infants and young children aged between 6-23 months in Fasting season.

Introduction

Dear how are you? My name is ………………….I am a member of data collector to collect data for the study conducted for the purpose of assessment of minimum acceptable diet among infants and young children. Next to this I want to ask some questions based on dietary diversity and meal frequency practice in Infants and young children in Fasting season. The result of this study will solve problems of feeding problems in Infants and children. Your correct answer for all questions is important to get good result. You can ask any questions if there is no clear question. The confidentiality is kept. I want to confirm that your name is not registered and mentioned at any time and any place. You participate in the study if and only if I get your permission. You have the right not to participate in the study or you can drop the questions if you are not comfortable with the questions. The interview will not take more than 30 minutes.

|  |
| --- |

Are you voluntary for interview? Yes I am voluntary.

|  |
| --- |

No I am not voluntary

Name of data collector ……………………...signature……………

Date of data collection ……………

Supervisor name and signature ………………………………. Code ……..

**Thank you for your participation.**

Part one

1. Socio- demographic factors of mother/caregivers

| Serial  Number | Questions | Answer |  |
| --- | --- | --- | --- |
| 1.1 | age | ………………… |  |
| 1.2 | Ethnicity | 1.Amhara  2.Oromo  3.Tigra  4 other specify |  |
| 1.3 | Current marital status | 1.single  2.married  3.divorced  4.died  5. separated |  |
| 1.4 | Mother educational level | 1. Can Read and Write  2.Primary school(1-8)  3.Secondary school (9-12)  4.college/ university  5 cannot read and write |  |
| 1.5 | Father’s educational level | 1. Cannot Read and Write  2.Primary school(1-8)  3.Secondary school (9-12)  4.college/ university  5. cannot read and write |  |
| 1.6 | your occupation | 1.housewive  2.daily worker  3.farmer  4.merchant  5. no current work  6.other specify |  |
| 1.7 | father’s occupation | 1.farmer  2.merchant  3.employed  4. daily worker  5.no job  6.other specify |  |
| 1.8 | Number of family members in the house including the child | Number |  |
| 1.9 | number of under 5 children live in the house | number |  |
| 1.10 | Sex of the child | 1. Male 2. female |  |
| 1.11 | age of infant or child | …………………… |  |
| 1.12 | child birth order |  |  |
| 1.13 | Have you television or radio? | 1.Yes 2.no |  |
| 1.14 | If you say yes for question 1.12 how many times you listen or watch television per a week? | times |  |

Part Two

Maternal health and child health service data

| S. no | Questions | Answer choice |
| --- | --- | --- |
| 2.1 | Did you have antenatal care follow up during pregnancy of the current child? | 1. yes  2. no |
| 2.2 | If the above question is yes how many times you got the service? | ………………….. |
| 2.3 | What was the place of delivery of the current child? | 1. Health facility  2. Home  3. Other specify…. |
| 2.4 | Have you history of PNC service after delivery of the current child? | 1. yes  2. no |
| 2.5 | If you say when you get the service after delivery? | 1.Within 1-2 day  2.Within 3-6 days  3.After 7 days |
| 2.6 | Is there any growth monitoring and promotion service given for the current child monthly? | 1.Yes  2. no |
| 2.7 | What is the vaccination status of the child? | 1. Not started 2. Up to date 3. finished |

Part Three

Breast feeding status, 24 hours recall Dietary diversity and meal frequency feeding practice

| S.no | Questions | Choice |
| --- | --- | --- |
| 3.1 | What is your child breast feeding status currently? | 1. Breast feed 2. Non breast feed |
| 3.2 | If the answer is 2, how many times the child consume milk for the last 24 hours? | Times |
| 3.3 | When you start additional food for a child? | 1. Before 6 months after delivery  2. At 6 months 3. After 6 months  4. I do not know |

The following questions contain food groups. It is prepared to assess 24 hours diet intake. Please circle 1 in answer column if the child consumes at least one food item in each group in the previous day and night and circle 0 if the child did not consume any type of foods listed in each food groups.

| s.no | Food groups | List of foods in each food groups | 1. Yes 0. No | |
| --- | --- | --- | --- | --- |
| 3.4 | Grains, roots and tubers | Barley, Maize, oat, sorghum, wheat, pasta, macaroni, bread, Enjera, potato, teff, porridge, dagusa, rice, and foods prepared from these food types. | 1 | 0 |
| 3.5 | Legumes and nuts | Bean, ocholone, guaya, , shimbra, peas, lentils, or nuts, or foods made from these | 1 | 0 |
| 3.6 | Dairy products (milk, yogurt, cheese) | Milk, cheese, yogurt, or other milk products | 1 | 0 |
| 3.7 | Flesh foods (meat, fish, poultry and liver/organ meats | Meat of, liver, beef, lamb, goat, chicken, | 1 | 0 |
| 3.8 | Egg | egg from chicken, any other egg | 1 | 0 |
| 3.9 | Vitamin-A rich fruits and vegetables | Carrot, orange, lettuce, pumpkin, cabbage, squash, ripe mangoes, ripe 1papayas, and 100%fruit juice made from these, or sweet potato, squash, | 1 | 0 |
| 3.10 | Other fruits and vegetables | Banana, tomato, avocado, lemon, peach, corn(fresh not dried, green maize, including, and 100% fruit juice made from these foods | 1 | 0 |

| 3.11 | Did your child consume Any oil, fats, or butter, or foods made with any of these for the last 24 hours? | | | 1 | 0 |
| --- | --- | --- | --- | --- | --- |
| 3.12 | Did your child consume any sugary foods such as chocolates, sweets, candies, pastries, cakes, or biscuits for the last 24 hours? | | | 1 | 0 |
| 3.13 | Did your child consume Condiments for flavor, such as chilies, spices, herbs, or fish powder? | | | 1 | 0 |
| 3.14 | Did your child consume foods made with red palm oil, red palm nut, and red palm nut pulp sauce for the last 24 hours? | | | 1 | 0 |
| 3.15 | List out the types of foods that the child consumed in the last 24 hours................. | | | | |
| 3.16 | | Do you know the appropriate frequency  Of feeding of your child per 24 hours? | 1.yes 2.no | | |
| 3.17 | | How do you compare the feeding frequency during non-fasting season from fasting season? | 1.similar  2.decrease at fasting  3.increase at fasting  4 I did not know | | |
| 3.18 | | If you say decrease during fasting season, why? | 1.fear of God because fasting day 2.restriction of food 3.because busy in work 4.because you remember the child feeding as other family member eat | | |
| 3.19 | | How many times the child consumed any soft, solid and semi-solid foods in the last 24 hours? | times | | |

**Part Four**

The following questions include other information which affects practice of dietary diversity and feeding frequency and answer the question by circle the answer you agree and if there is specify write the answer on place provided at the end of each row of a question.

| Serial no. | Question | Answer code |
| --- | --- | --- |
| 4.1 | Have you read magazine, newspaper? | 1.Yes 2.No |
| 4.2 | If you say yes for question 4.1 how many times you read per a week? | 1.One times  2. Two times  3.Three times and above |
| 4.3 | Do you know that the child should take diversified diet at any time? | 1.Yes  2. no |
| 4.4 | Do you believe that the child should not take animal products during fasting season? | 1.yes, I believe  2. no, I do not believe |
| 4.5 | If you say, why? | 1. The food item is not necessarily for the child at this age.  2. The food item may mix with other foods.  3. The child should not take it because it is fasting season and fear of GOD. |
| 4.6 | If you are recommended to give animal products to the child at this time by the following people at different time and place, whose recommendation will be implemented? | 1.health extension workers  2.health worker from health centers  3.religion leaders  4.husband/ wife  5.kebele administrator  6. Other specify… |
| 4.7 | If you are given an advice to feed the child with appropriate frequency based on the age by the following people whose advice is selected? | 1.health extension workers  2.health worker from health centers  3.religion leaders  4.husband/ wife  5.kebele administrator  6. Other specify |
| 4.8 | Is there discussion on Infant and young child feeding practice on female health development army meeting? | 1.yes  2.no |
| 4.9 | If you say yes for the above question, what agreement was shared by the health development army on feeding infants and young child with animal products and meal frequency during fating and non-fasting season? | 1. always similar feeding practice  2.not give animal product during fasting  3.dcrease feeding frequency during fasting |
| 4.10 | Is there any education or information you get on the Importance of feed the child with diversified food and feeding frequency? | 1=yes  2= no |
| 4.11 | If you say yes for question 4.10 by what means you get Information and education? | 1. television/radio  2.friend  3.family member  4.health professionals  5.relative  6. religion leaders  7. Other specify…… |
| 4.12 | Did you get practical observation of training on food preparation with different food items? | 1=yes  2=no |
| 4.13 | If you say yes for question number 4.10 where you get? | 1.television  2.health professionals  3.friends  4. other specify |
| 4.14 | Who give a decision role in the household? | 1.mother  2.father  3.other specify |
| 4.15 | Is there any family member who eats any food item including animal products for the last 24 hour other than the child? | 1=yes  2=no |
| 4.16 | Is there any family member who eats at any time without considering fasting time during 24 hours other than the child? | 1=yes  2=no |
| 4.17 | Is there any information given to you by religion leaders on no need of child feeding with animal products? | 1=yes  2=no |
| 4.18 | Is there any information given to you by religion leaders on feeding of child with less frequency as non-fasting season? | 1=yes  2=no |
| 4. 19 | Do religion leaders give education on child feeding at church or other place? | 1. Yes 2. no |

**Part five: Wealth index related characteristics**

| **S.no.** | **Questions** | **Alternatives** |
| --- | --- | --- |
| 5.1 | Ownership of the house | 1. Private  2. Rented from individual  3. Others (specify)________ |
| 5.2 | How many rooms are there in your home? | __________in number |
| 5.3 | What is the main material of the dwelling floor? | 1. Earth / Sand  2. Cement  3. Bamboo  4. Carpet  5. Others (specify)_________ |
| 5.4 | What is the main material of the roof? | 1. Iron corrugated sheet 2. Wood  3. Thatch 4. Bamboo  5. Others (specify)_________ |
| 5.5 | What is the main material of the exterior walls? | 1. Stone with mud 2.Wood with mud   3. Stone with cement  4. Others (specify)_________ |
| 5.6 | What type of fuel mainly used for household cooking? | 1.Electricity 2.Charcoal 3.Wood 4.Animal dung  5.Others (specify)___ |
| 132 | Is the cooking usually done in the house, in a separate building, or outdoors? | 1. In a separate room used as kitchen  2. Elsewhere in the house  3. In a separate building  4. Other *(*specify*)________* |
| 133 | How many hector of agricultural land do you have? | ______________ |
| 134 | How many hector of irrigation land do you have? | ______________ |
| 135 | Annual total agricultural products(includes all items) | ___________quintal |
| 136 | Does your household have   1. Electricity? 2. A Radio? 3. A Television? 4. A Non-mobile telephone? 5. A Refrigerator? 6. Table? 7. Chair? 8. A bed with cotton/spring mattress | \| Yes \| No \| \| --- \| --- \| \| 1 \| 2 \| \| 1 \| 2 \| \| 1 \| 2 \| \| 1 \| 2 \| \| 1 \| 2 \| \| 1 \| 2 \| \| 1 \| 2 \| \| 1 \| 2 \| |
| 137 | Does any member of your household own   1. A watch? 2. A mobile phone? 3. A bicycle? 4. A Bajaj? 5. Animal drawn cart? 6. Car? | \| Yes \| No \| \| --- \| --- \| \| 1 \| 2 \| \| 1 \| 2 \| \| 1 \| 2 \| \| 1 \| 2 \| \| 1 \| 2 \| \| 1 \| 2 \| |
| 138 | Does this household own any livestock, herds, other farm animals, or poultry? | 1. Yes 2. No |
|  | How many of the following animals do the household have? |  |
|  | 1. Cattle, milk cows, bulls? | _________in number |
|  | 1. Horses, Donkeys, or mules? | _________in number |
|  | 1. Goats? | _________in number |
|  | 1. Sheep? | _________in number |
|  | 1. Chickens? | _________in number |
|  | 1. Beehives? | _________in number |
| 139 | Do you have Bank account or Amhara credit and saving institution? | 1. Yes  2. No |
| 140 | If yes Q139, how much money do you have in the bank or Amhara credit and saving institution? | ____________ETB |
| 141 | Where do you access drinking water | 1. Tape water 2. Protected spring 3. Unprotected spring/river |
| 142 | Do you have a latrine? | 1. Yes 2. No |
| 143 | What type of latrine facility do you have? | 1. Flush to piped sewer system 2. Flush to septic tank 3. Ventilated improved pit (VIP) latrine 4. Pit latrine without VIP a slab 5. Open field |
| 5.20 | What source of light you use as a source of light at night? | 1. Animal dung 2. wood  3. kuraz 4 other specify |
